# Pain Hypersensitivity and Increased Urinary Tetrahydrobiopterin Levels in Mice Submitted to High-Fat Diet

**DOI:** 10.3390/brainsci15060646

**Published:** 2025-06-16

**Authors:** Tuany Eichwald, Débora da Luz Scheffer, Leonardo Barros, Alexandre Solano, Vivian De Souza Menegassi, Ananda Christina Staats Pires, Camila Sartor Spivakoski, Rodrigo A. Da Silva, Joana M. Gaspar, Marcelo Fernando Ronsoni, Alexandra Latini

**Affiliations:** 1 Laboratory of Bioenergetics and Oxidative Stress (LABOX), Department of Biochemistry, Federal University of Santa Catarina, Florianópolis 88037-100, Brazil; 2 Department of Gastroenterology, Health Sciences Center, University Hospital, Federal University of Santa Catarina, Florianópolis 88037-100, Brazil; 3 Department of Internal Medicine, Health Sciences Center, University Hospital, Federal University of Santa Catarina, Florianópolis 88037-100, Brazil; 4 Epigenetic Study Center and Gene Regulation (CEEpiRG), Program in Environmental and Experimental Pathology, Paulista University (UNIP), São Paulo 05508-070, Brazil; 5 School of Dentistry, University of Taubaté, Taubaté 12020-3400, Brazil

**Keywords:** obesity, adipose tissue, physical exercise, chronic pain, biomarker

## Abstract

**Objective:** This study aimed to examine the impact of high-fat-diet(HFD)-induced obesity on pain sensitivity and tetrahydrobiopterin (BH4) levels. The effect of moderate-intensity physical exercise, an anti-inflammatory non-pharmacological intervention, on pain scores was also investigated. **Methods**: Adult male C57BL/J6 mice were fed standard or an HFD for eight weeks. Their total body weight, food intake, locomotor and motivational behavior, and pain reflexes were measured. A subgroup of animals underwent physical exercise for five days per week over six weeks. Blood was collected for glucose tolerance testing and levels of lactate. Urine samples were collected to measure BH4 levels. **Results**: We showed that the HFD increased weight gain, epididymal white adipose tissue, and the percentage of body weight as epididymal fat. These anthropometric alterations were characterized by impaired glucose tolerance at four and eight weeks of the dietary intervention. It was also observed that reduced locomotor activity and higher pain scores in the HFD-fed mice were prevented by a physical exercise intervention. The HFD also induced an increase in urinary BH4 levels at four and eight weeks of intervention. **Conclusions**: The HFD increased scores of chemical and mechanical hyperalgesias, as well as urinary BH4 levels. Urinary BH4 can be proposed as a potential easy-to-access, sensitive, and reliable biomarker of pain development, and a promising target for the control of pain hypersensitivity in obesity.

## 1. Introduction

Obesity is defined by the World Health Organization (WHO) as abnormal and excessive accumulation of fat [1]. The increase in white adipose tissue (WAT) from a daily positive energy balance is associated with low-grade chronic systemic inflammation, with immune infiltration in the WAT. As of 2022, more than 1 billion people worldwide are living with obesity—650 million adults, 340 million adolescents, and 39 million children [1]. In Brazil, the prevalence of obesity among adults increased significantly from 11.8% in 2006 to 24.3% in 2023, representing a 12.5 percentage point rise—an approximate relative increase of 105.9% over the period [2]. Obesity is a multifactorial etiology disease, and the clinical manifestations involve chronic pain and joint dysfunction [3]. Obesity predisposes the development of many chronic diseases, including inflammatory and degenerative conditions of the musculoskeletal system and the peripheral and central nervous systems [4,5]. Indeed, the inflammatory pathways are persistently activated in several brain regions controlling peripheral regulation of energy, glucose, and lipid metabolism [6,7].

WAT functions as a dynamic endocrine organ, playing a central role in the regulation of metabolic and inflammatory processes. WAT secretes various pro-inflammatory adipokines, such as tumor necrosis factor alpha (TNF-α), interleukin (IL)-6, IL-18, and leptin, and its expansion disrupts the balance of inflammatory mediators by reducing anti-inflammatory factors, thereby promoting a chronic low-grade systemic inflammatory state [8]. Importantly, central adiposity is strongly associated with sedentary behavior, whereas regular physical activity has been shown to counteract WAT inflammation and reduce the risk of obesity-related metabolic dysfunctions [9]. The anti-inflammatory benefits of physical exercise are closely linked to its duration and intensity. For instance, longer training sessions are associated with a greater reduction in WAT [10]. Engaging in regular physical activity has been recommended as a non-pharmacological approach for managing various pain-related conditions, showing clinical outcomes comparable to those achieved with pharmacological drugs [11].

Tetrahydrobiopterin (BH4) is a pterin that acts biologically as a mandatory cofactor for the metabolism of phenylalanine and certain lipids, and for the biosynthesis of the neurotransmitters dopamine, serotonin, and nitric oxide (NO) [12,13]. BH4 intracellular concentrations are finely tuned by three metabolic pathways, assuring continuous basal levels of the molecule to support the systems where it is involved (see [14] for a review). However, excessively increased BH4 levels have been associated with numerous chronic inflammatory pathological conditions, including cardiovascular disease [15], cancer [16], and chronic pain [17,18], among others. Furthermore, pro-inflammatory and oxidant mediators, such as IL-1β, TNF-α, interferon gamma (IFN-γ), and hydrogen peroxide, positively enhance the synthesis of BH4 in immune, central, and peripheral nerve cells, and others [19,20].

Pain is a conscious experience that demands cortical participation and aversive information from nociception, processed by the peripheral nervous system, and unconsciously modulated by the central nervous system [21]. Normally, adaptive responses to stimuli activating both systems effectively overcome the threat and lead to resolution. However, maladaptive inflammatory reactions, in which pro-inflammatory mediators persistently activate and sensitize neurons at different levels of the nociceptive pathway, are believed to induce chronic pain [8,22]. Thus, this work aimed to identify whether obesity, a low-degree chronic inflammatory disease, may negatively modulate nociceptive thresholds and positively increase BH4 levels that can be monitored in biological fluids as a biomarker for pain, and to investigate whether moderate-intensity physical exercise could prevent these obesity-related effects.

## 2. Materials and Methods

### 2.1. Animals

Adult male C57BL/J6 mice (3–5 months of age; 25–30 g) from the Centre for Biological Sciences, Universidade Federal de Santa Catarina (UFSC) (Brazil) were acclimated for ten days in a controlled environment (22 ± 1 °C, 12 h light/dark cycle) with free access to water and food. All experimental protocols, approved by UFSC’s Ethics Committee for Animal Research (CEUA, 4401201118), complied with current guidelines for laboratory animals and ethical care and ethical standards for experimental pain research in conscious animals [23].

### 2.2. Experimental Strategy

**Scheme 1:**
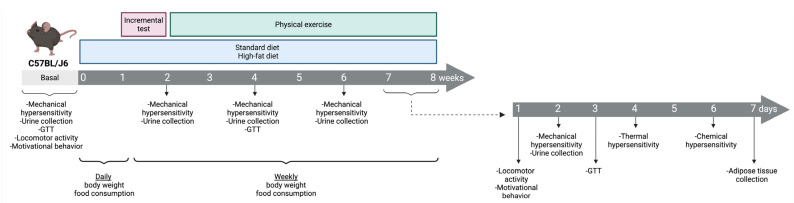
**Schematic representation of the experimental design using C57BL/6 mice.** The mice were subjected to either a standard diet or a high-fat diet for 8 weeks, with a concurrent physical exercise regimen for 6 weeks. An incremental test was performed at the beginning of the study. Throughout the 8-week period, body weight and food consumption were recorded daily during the first week and weekly in the following 7 weeks. Behavioral and physiological assessments, including urine collection, glucose tolerance test (GTT), locomotor activity, motivational behavior, and mechanical, thermal, and chemical hypersensitivity testing, were conducted at specific time points. After 8 weeks, the study concluded with epididymal adipose tissue collection.

Mice were randomly divided into two groups. One group received a high-fat diet (HFD), while the other group received a standard diet. Both groups were maintained on their respective diets for 8 weeks [24]. The mice were fed with HFD (n = 13) to induce increased adiposity and to be used as a proxy of human obesity. The diet’s macronutrient composition was proteins: 12 kJ% (19 g%); carbohydrates: 27 kJ% (45 g%); and lipids: 61 kJ% (36 g%) (Pragsoluções Biociências, Jaú, São Paulo, Brazil) [25]. The animals fed with the standard diet were used as controls (n = 12). The standard diet composition was proteins: 20 kJ% (24 g%); carbohydrates: 65 kJ% (63 g%); and lipids: 15 kJ% (13 g%). The animals were housed in cages containing 4–5 individuals from the same experimental group. The cages were labeled with the type of treatment (diets), and the corresponding food was provided. During the whole experimental strategy, the mice were closely monitored for humane endpoints. To prevent HFD-induced WAT accumulation, a group of animals was also submitted to physical exercise, as described below. Basal measurements, including thermal hypersensitivity, urine collection, glucose tolerance test (GTT), locomotor activity, and motivational behavior, were performed on four separate days during the week preceding the dietary intervention. The dietary intervention started on the Monday of week 0 and continued for 8 weeks, ending on a Monday, at which point the animals were euthanized for sample collection and analysis. In the final (eighth) week, measurements were conducted across seven consecutive days, from Monday to the following Monday, to capture endpoint assessments. The complete experimental protocol is depicted in Scheme 1.

Prior to the start of the interventions, all the mice underwent measurements as part of a “baseline” group. This group refers to all the animals evaluated before experimental group allocation, when they were still maintained under identical conditions, receiving the standard diet. These baseline measurements served as reference values before the initiation of the dietary and physical activity interventions.

The experimental groups consisted of n = 6–7 mice. This number allowed for the performance of the behavioral tests with sensitivity and reproducibility, and to collect tissues generating an appropriate unit of mass to perform the biochemical measurements. This number of sample size was calculated by applying “power statistics”, as shown below [26].

Sample size calculation: We used the following formula for sample size calculation for the comparison between two groups with quantitative data endpoints: sample size = 2 SD^2^ (Z^a/2^ + Z^β^)^2^/d^2^.

Where the Standard deviation (SD) = estimated from previous studies:

Z^a/2^ = Z_0.05/2_ = Z_0.025_ = 1.96 (From Z table) at type 1 error of 5%

Z^β^ = Z 0.20 = 0.842 (From Z table) at 80% power

d = effect size = difference between mean values

Hence, now the formula will be:

n = 2⋅SD^2^⋅(Z_1 − α_/2 + Z_1 − β_)2d^2^n = d22⋅SD^2^⋅(Z_1 − α_/2 + Z_1 − β_)2

Based on previous studies from our group, we have the following assumptions:

- The minimum difference between the groups’ means will be set at least at 54.4%;
- Biological experiments inherently have a 10–15% error margin;
- Differences less than 20% in each group’s mean can increase the probability of type I or type II errors;
- The standard deviation is typically 35% of the mean value.

Sample size = n = 2·(35)2·(1.96 + 0.842)2(55)2 ≈ 7n = (55)22·(35)2·(1.96 + 0.842)2 ≈ 7.

### 2.3. Calorie Intake Measurement

During the first week of the experimental protocol, food consumption was measured daily, while in the subsequent seven weeks, it was measured once per week. To determine intake, the amount of food (in grams) provided to each cage was recorded, and the remaining food was weighed at each time point (daily in the first week and weekly in the seven subsequent weeks). The difference was considered the amount consumed. This value was then divided by the number of animals in the cage to estimate the average food consumption per animal. Based on the known caloric density of each diet (kcal per 100 g), the calorie intake was calculated by multiplying the estimated gram-based consumption per animal by the corresponding energy content of the diet and dividing by 100 (100 g of diet).

### 2.4. Intraperitoneal Glucose Tolerance Test

The glucose tolerance test (GTT) was performed after 6 h of fasting by injecting the mice with 2 g/kg glucose intraperitoneally (i.p.). Blood glucose levels were measured at 0, 5, 10, 15, 30, 60, and 120 min from tail blood samples (adapted from [27]), to capture early glucose absorption and peak glycemia, and later clearance. The test was performed before the dietary intervention, at 4 and 8 weeks.

### 2.5. Physical Exercise

#### 2.5.1. Incremental Test to Determine the Maximal Capacity for Exercise

The incremental test was performed to identify 60% of the maximal capacity of the animals [28]. When exhaustion was reached, caudal blood was collected to measure lactate levels.

A motorized treadmill was adapted to simulate running exercise in mice. The treadmill’s pulley and motor were modified to allow a minimum speed of 5 m/min, and the running surface was adjusted to remain horizontally leveled. An acrylic apparatus containing 12 individual lanes (bays) was attached to the treadmill to allow simultaneous but separated exercise of multiple animals [28]. During the first week of training, performance was scored daily: 1 for animals that refused to run; 2 for animals that ran at variable speeds, ran, and stopped; 3 for animals that ran regularly; 4 for animals that were runners; and 5 for animals that were good runners. Only animals that scored 3 or more continued the training. Two animals scored less than 3 and were discontinued from the physical exercise protocol.

#### 2.5.2. Physical Exercise Protocol

The physical exercise protocol consisted of five training sessions/week for 6 weeks on a treadmill with an interval of 48 h each week (Table 1) [28]. The animals began with a 5 min warm-up at 40% intensity before each session. The training speed was set at 60% of the final speed from the incremental test, indicating moderate intensity. The sessions lasted 35, 40, and 45 min/day for the first 3 weeks, with a subsequent incremental test. The last 2 weeks’ sessions lasted 35 and 40 min/day [29].

##### Caudal Blood Collection

Immediately at the end of the incremental test, 50 μL of caudal blood was collected in tubes containing sodium fluoride to inhibit glycolysis [30]. The blood was used to measure lactate concentrations.

##### Lactate Measurement

The lactate levels were measured using the YSI Biochemistry Analyzer (YSI Inc., Yellow Springs, OH, USA) equipped with a specific lactate sensor, as previously published by our group [31]. High blood lactate indicated the exercise protocol’s intensity.

### 2.6. Behavioral Tests

The animals were acclimated for 1 h before behavioral tasks in the experimental room. The assessments occurred during the rodent’s light phase and were conducted by a researcher blinded to the experimental groups.

#### 2.6.1. Locomotor Activity

Locomotor activity was evaluated in a 100 cm × 100 cm × 50 cm open field arena in a sound-attenuated room under low-intensity light. Each animal’s exploratory activity was video recorded for 5 min and analyzed using the ANY-mazy Platform^TM^ version 7.49 [32]. The animals were evaluated before and after the dietary intervention.

#### 2.6.2. Motivational Behavior

Motivational behavior was assessed by measuring the grooming behavior for 5 min after mice were squirted with 1 mL of a 10% sucrose solution on their dorsal coat [33]. Due to its viscosity, the sucrose solution sticks to the animal’s hair, inducing a grooming behavior. The frequency (duration) and the latency to the beginning of the grooming behavior were observed for 5 min. The animals were assessed before and after the dietary intervention.

#### 2.6.3. Mechanical Hypersensitivity

To assess mechanical sensitivity, the withdrawal threshold was measured using a series of von Frey filaments (0.20, 0.40, 0.70, 1.6, 3.9, 5.9, 9.8, and 13.7 mN, Stoelting, Wood Dale, IL, USA; equivalent in grams to 0.02, 0.04, 0.07, 0.16, 0.40, 0.60, 1.0, and 1.4). The 50% withdrawal threshold was determined using the ‘up-down’ method and calculated using the Up-Down Reader software version v2.0 [34]. The animals were evaluated before and 2, 4, 6, and 8 weeks after the dietary intervention.

#### 2.6.4. Thermal Hypersensitivity

The mice were placed in an acrylic cylinder on the surface of a previously heated metal plate (52 ± 0.5 °C) (INSIGHT^®^, Ribeirão Preto, Brazil) [18]. The latency that the animal took to stand up, shake, and/or lick one of the hind legs was considered an indication of their thermal hypersensitivity [35]. The animals were evaluated after 8 weeks of dietary intervention.

#### 2.6.5. Chemical Hypersensitivity

The animals received a 20 µL injection containing 1.6 µg capsaicin under the skin on the dorsal right hind paw. The animal was then placed in a transparent glass chamber and observed for 5 min. The latency and the number of times of the licking and/or shaking of the paw, fingers, or leg where the capsaicin was injected were timed [36]. The animals were assessed after the dietary intervention.

### 2.7. Urine Sample Collection

Urine samples were spontaneously collected from the animals during handling prior to placement in the von Frey apparatus. The urine was collected non-invasively during spontaneous urination, without applying any form of physical restraint, as previously published [37]. The urine was immediately captured onto parafilm and transferred into 1.5 mL tubes. The samples were collected before and 2, 4, 6, and 8 weeks after the dietary intervention.

### 2.8. Adipose Tissue Dissection

The animals were euthanized at the end of 8 weeks of dietary intervention using cervical dislocation and following the ARRIVE guidelines, to collect blood and epididymal WAT (eWAT).

### 2.9. BH4 Quantification

Urinary BH4 levels were determined by high-performance liquid chromatography (HPLC) (Alliance e2695, Waters, MA, USA) coupled with electrochemical detection, as previously described with some modifications [33]. The results were expressed as μmol/mmol of creatinine.

#### Quantification of Creatinine

The urine creatinine concentrations were determined using a commercial kit based on the Jaffé reaction (Pointe Scientific Inc., Canton, MI, USA), according to the manufacturer’s instructions. The urine samples were diluted 1:10 in distilled water prior to analysis. The creatinine levels were indicated as mmol/L.

### 2.10. Gene Promoter DNA Methylation

For the determination of DNA methylation of the promoters for genes involved in tetrahydrobiopterin (BH4) biosynthesis, first, the genomic DNA (gDNA) of the hippocampus was isolated using the phenol/chloroform/isoamyl alcohol method and subsequently digested with the restriction enzymes MspI or HpaII (R0106L, New England BioLabs, Ipswich, MA, USA) [38]. Following DNA treatment, qPCR reactions were performed in 40 amplification cycles in a total volume of 10 μL, containing PowerUp™ SYBR™ Green Master Mix 2 × (5 μL; A25741, Applied Biosystems, Carlsbad, CA, USA), 0.5 μM of each primer [Dhfr (F: 3′ CGC CTA GGT GGA CGC TAG GGG C5′, R: 3′ CTT AGC TAC ACA CAA ATA GAA TGC 5′), Spr (F:3′ AGC ACC ACT TTC AGG TCA GG 5′, R: 3′ ACT CAT GCG CAG CTA CTG AA 5′), Ptps (F: 3′ CAC GTG GGC ACA CCC CAC CT 5′, R: 3′ GAG TGG TTT CGA CTC GCG GCA CCC TTC 5′)], 1 μL of treated gDNA, and nuclease-free water.

### 2.11. Statistical Analysis

The data are presented as the mean ± standard error of the mean. The data were analyzed by one-way or two-way ANOVA, followed by the post hoc tests of Šídák or Tukey when *F* was significant. When comparing the two independent groups, the one-tailed Student’s *t* test was used. The accepted level of significance for the tests was *p* ≤ 0.05. Statistics and all graphs were performed by using GraphPad Prism 9^®^. The outlier test was performed using GraphPad Prism, employing the ROUT (Robust Regression and Outlier Removal) method to identify and exclude statistical outliers.

## 3. Results

**Figure 1:**
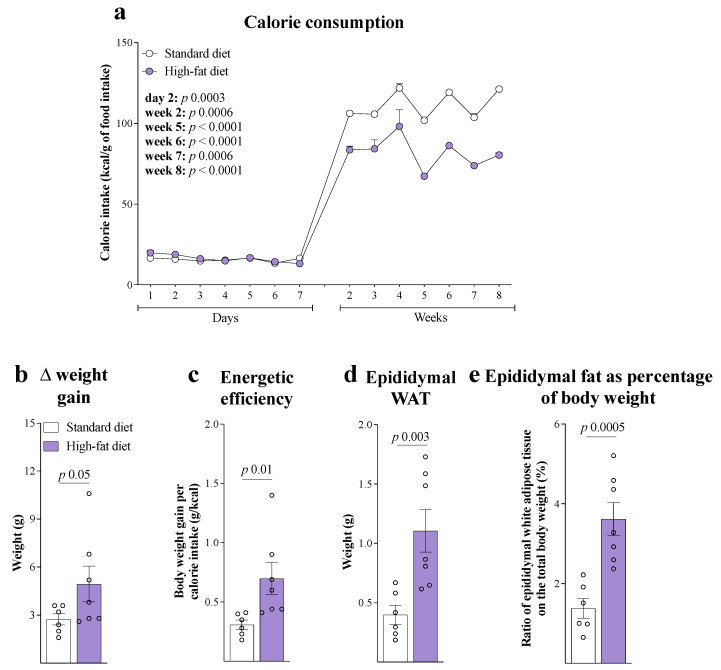
**Epididymal white adipose tissue (WAT) accumulation and higher body fat in C57BL/J6 mice submitted to the high-fat diet (HFD).** Adult male C57BL/J6 mice received the HFD ad libitum for 8 consecutive weeks (proteins: 12 kJ% (19 g%); carbohydrates: 27 kJ% (45 g%); lipids: 61 kJ% (36 g%)). The controls received a standard rodent diet for the same period (20 kJ% (24 g%); carbohydrates: 65 kJ% (63 g%); lipids: 15 kJ% (13 g%)). (**a**) Daily calorie intake. (**b**) The delta of weight gain between the final weight and the initial weight. (**c**) The energy efficiency, calculated by dividing the energy content (in kcal) by the weight of the foods (in g) consumed. (**d**) Accumulated epididymal WAT after 8 weeks of intervention. (**e**) The relationship between epididymal WAT and body weight. ANOVA for repeated measures, followed by the Šídák post hoc test for multiple comparisons for (**a**) the one-tailed Student’s *t*-test for (**b**–**e**). (n = 6–7 animals per group).

Figure 1 shows the effect of the HFD on caloric consumption, body weight, and fat accumulation. Figure 1a shows that caloric consumption was significantly lower in the animals submitted to the HFD compared to the animals that received the standard diet. However, Figure 1b shows that the weight gain delta, calculated as the final body weight minus the initial body weight, was increased in the HFD-fed mice [*t*_(11)_ = 1.77; *p* ≤ 0.05]. In agreement, the energy efficiency of the diets (Figure 1c) was shown to be higher in HFD-fed mice [*t*_(11)_ = 2.28; *p* < 0.05]. Figure 1d shows that the weight of the eWAT was significantly higher in the HFD-fed animals [*t*_(11)_ = 3.38; *p* < 0.01]. Similarly, the relationship between eWAT and the total body weight was increased in the group of animals fed with the HFD (Figure 1e) [*t*_(11)_ = 4.42; *p* < 0.001]. However, no significant differences were observed in the total body weight between the groups throughout the 8-week intervention period (Supplementary Figure S1). Supplementary Figure S2A,B are representative images taken from a mouse from each experimental group.

**Figure 2:**
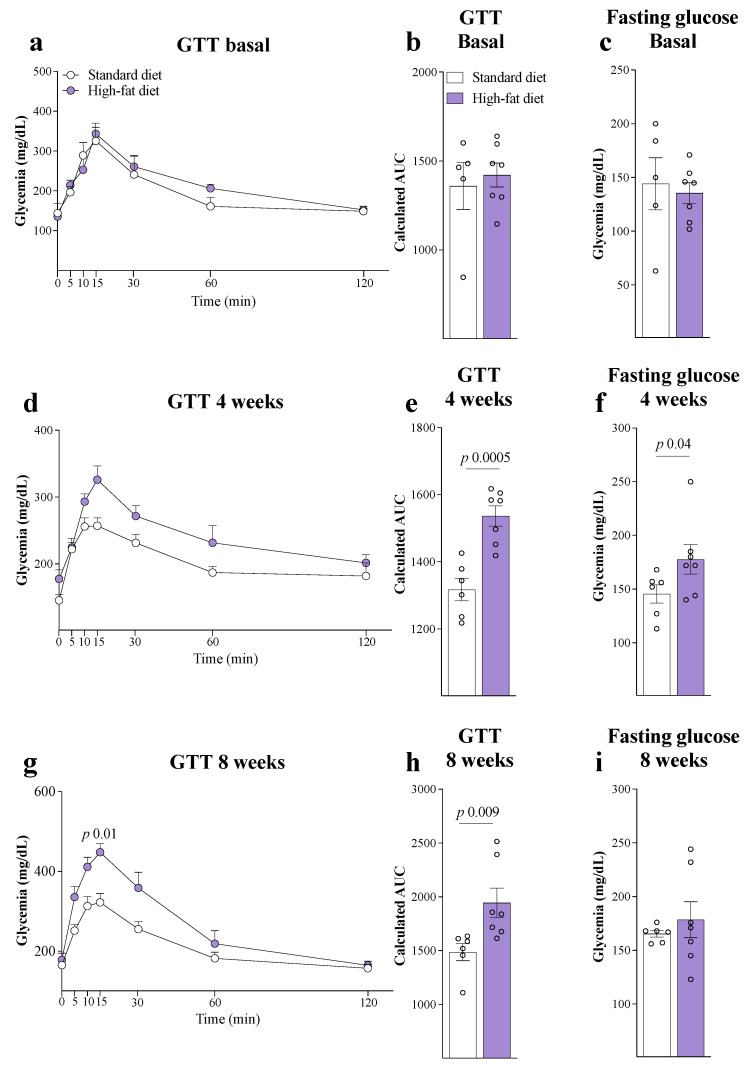
**The glucose tolerance test (GTT) impairment in C57BL/J6 mice submitted to the high-fat diet (HFD).** Adult male C57BL/J6 mice received the HFD ad libitum for 8 consecutive weeks (proteins: 12 kJ% (19 g%); carbohydrates: 27 kJ% (45 g%); lipids: 61 kJ% (36 g%)). The controls received a standard diet for the same period (20 kJ% (24 g%); carbohydrates: 65 kJ% (63 g%); lipids: 15 kJ% (13 g%)). (**a**,**d**,**g**) The glucose tolerance test (GTT), (**b**,**e**,**h**) area under the curve of the GTT, and (**c**,**f**,**i**) fasting glucose were measured before the intervention, and at 4 and 8 weeks afterwards, respectively. ANOVA for repeated measures followed by the Šídák post hoc test for multiple comparisons for (**a**,**d**,**g**) the one-tailed Student’s *t*-test for (**b**,**c**,**e**–**g**,**i**). (n = 6–7 animals per group).

The GTT and the fasting glucose test were performed at three different times, baseline (pre-intervention), 4, and 8 weeks after intervention, to assess glucose tolerance and infer potential changes in insulin sensitivity (Figure 2). Figure 2a shows that the GTT was identical in both experimental groups before the dietary intervention. The area under the curve (Figure 2b) and fasting glucose levels (Figure 2c) were also not different. Figure 2d shows that the GTT was not altered after 4 weeks of intervention; however, the area under the curve shown in Figure 2e was significantly bigger in the HFD group [*t*_(11)_ = 4.90; *p* < 0.001], as well as fasting blood glucose levels (Figure 2f) [*t*_(11)_ = 1.903; *p* ≤ 0.05]. Figure 2g shows a significant increase in glycemia at 15 min [F_(6,66)_ = 4.68; *p* < 0.001], and the values under the curve [*t*_(11)_ = 2.774; *p* < 0.01] (Figure 2h) after the glucose injection in the HFD-fed mice. However, the fasting glycemia at week 8 (Figure 2i) was not statistically different between the groups.

**Figure 3:**
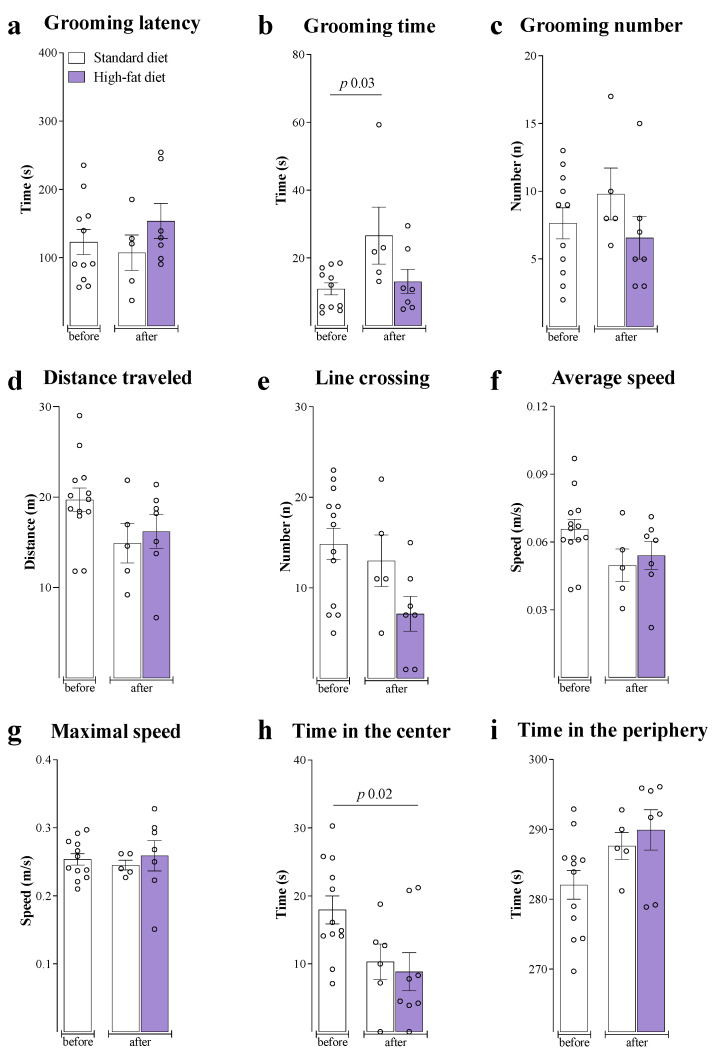
**The motivational behavior and spontaneous locomotor activity were not altered in C57BL/J6 mice submitted to the high-fat diet (HFD).** Adult male C57BL/J6 mice received the HFD ad libitum for 8 consecutive weeks (proteins: 12 kJ% (19 g%); carbohydrates: 27 kJ% (45 g%); lipids: 61 kJ% (36 g%)). The controls received a standard rodent diet for the same period (20 kJ% (24 g%); carbohydrates: 65 kJ% (63 g%); lipids: 15 kJ% (13 g%)). (**a**) The latency, (**b**) total time, and (**c**) number of grooming events evoked by spraying sucrose on the back. (**d**) The total distance traveled, (**e**) the number of intersections, (**f**) the average speed, (**g**) the maximum speed, (**h**) the time spent in the center of the apparatus, and (**i**) the time spent on the periphery of the apparatus. One-way ANOVA followed by Tukey’s post hoc test. (n = 5–11 animals per group).

Figure 3 shows the influence of the HFD on the motivational behavior and spontaneous locomotor activity. The “before” group refers to the baseline condition, in which all animals were housed under identical conditions and maintained on a standard diet prior to experimental group allocation. Figure 3a,c show that the performance in the sucrose test, used to assess motivation, depression, and anhedonia (Figure 3a), total time spent in grooming (Figure 3b), and number of grooming activities (Figure 3c) were not altered in the HFD-fed mice. However, the grooming total time was higher in the control group after 8 weeks of intervention [*F*_(2,20)_ = 0.80; *p* ≤ 0.05] (Figure 3b). To identify whether the diet would cause changes in locomotor activity, the open field test was performed before the intervention and at the end of 8 weeks of experimentation. Figure 3h shows that animals fed with the HFD for 8 weeks spent less time in the center of the apparatus [*F*_(2,23)_ = 0.11; *p* ≤ 0.05]. No significant differences were observed between the groups in the total distance traveled (Figure 3d), crossing (Figure 3e), average speed (Figure 3f), maximal speed (Figure 3g), and time in the periphery (Figure 3i).

**Figure 4:**
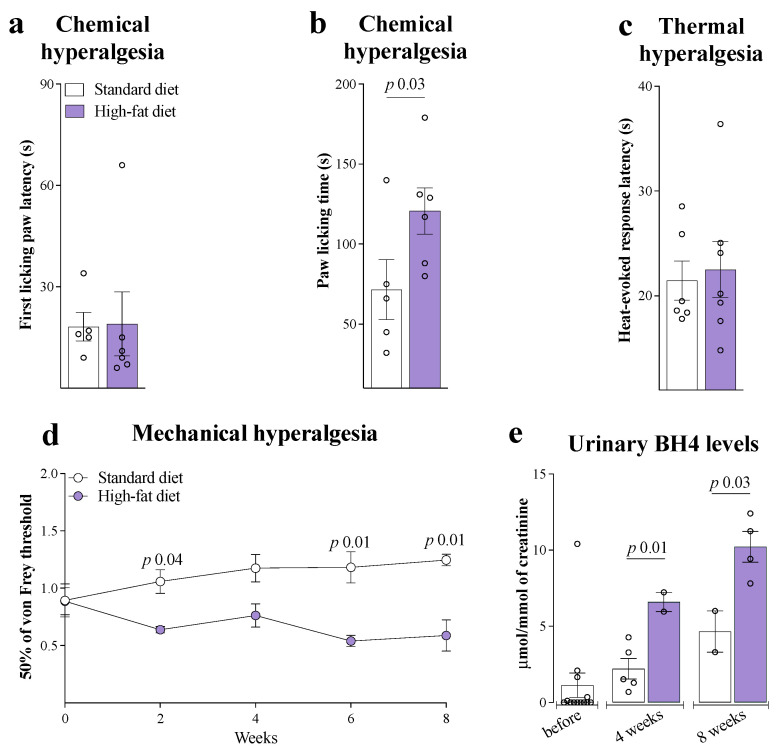
**Induction of hyperalgesia and increased urinary tetrahydrobiopterin (BH4) levels in C57BL/J6 mice submitted to the high-fat diet (HFD)**. Adult male C57BL/J6 mice received the HFD ad libitum for 8 consecutive weeks (proteins: 12 kJ% (19 g%); carbohydrates: 27 kJ% (45 g%); lipids: 61 kJ% (36 g%)). The controls received a standard diet for the same period (20 kJ% (24 g%); carbohydrates: 65 kJ% (63 g%); lipids: 15 kJ% (13 g%)). (**a**) The first response latency and (**b**) the total response time evoked by hind paw subcutaneous injection of capsaicin. (**c**) The latency for the first response evoked by the heat of the hot plate at 50 °C. (**d**) von Frey threshold used to determine mechanical hyperalgesia evaluated before and at weeks 2, 4, 6, and 8 after the intervention. (**e**) The BH4 concentrations in the urine before and at 4 and 8 weeks after the intervention. ANOVA for repeated measures followed by the Šídák post hoc test for multiple comparisons for (**d**) the one-tailed Student’s *t*-test for (**a**–**c**) and (**e**). (n = 5–7 animals per group).

To investigate whether the HFD would induce nociceptive changes under different noxious stimuli, the mice were evaluated for chemical, thermal, and mechanical hyperalgesia. Figure 4a shows that there was no difference between the groups in the latency for the animals’ first response evoked by capsaicin. However, the total response time evoked by capsaicin was higher in the animals fed with the HFD [*t*_(9)_ = 2.11; *p* ≤ 0.05] after the 8-week dietary intervention (Figure 4b). Figure 4c shows that there was no significant difference in the reflexes evoked by the thermal stimulus after the 8-week dietary intervention. The HFD-fed mice showed lower thresholds for mechanical hyperalgesia at weeks 2, 6, and 8 post-intervention (Figure 4d). To investigate whether BH4 levels were increased under the reduction in nociceptive thresholds, the levels of pterin were measured in the urine of the animals. Figure 4e shows higher levels of BH4 in the urine of the HFD-fed animals at 4 weeks [*t*_(5)_ = 3.73; *p* < 0.01] and 8 weeks [*t*_(4)_ = 3.32; *p* < 0.01] after the dietary intervention.

**Figure 5:**
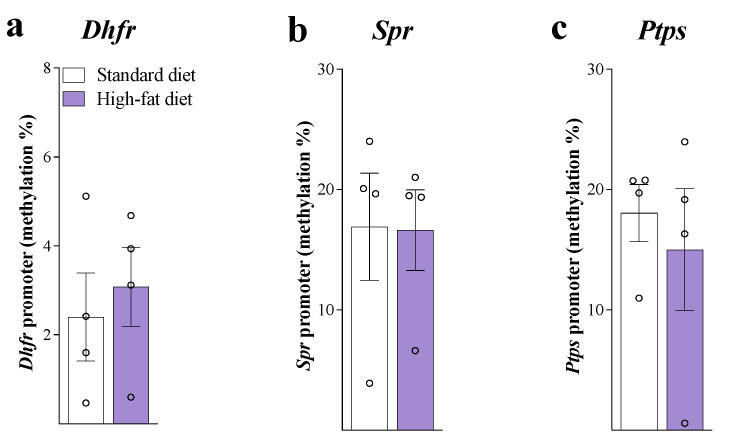
**The DNA methylation of the promoters for the genes involved in tetrahydrobiopterin (BH4) biosynthesis was not altered in C57BL/J6 mice submitted to the high-fat diet (HFD).** Adult male C57BL/J6 mice received the HFD ad libitum for 8 consecutive weeks (proteins: 12 kJ% (19 g%); carbohydrates: 27 kJ% (45 g%); lipids: 61 kJ% (36 g%)). The controls received a standard rodent diet for the same period (20 kJ% (24 g%); carbohydrates: 65 kJ% (63 g%); lipids: 15 kJ% (13 g%)). The percentage of promoter methylation for (**a**) *Dhfr*, (**b**) *Spr*, and (**c**) *Ptps* was measured after 8 weeks on the HFD. The one-tailed Student’s *t*-test (n = 4 animals per group).

To verify whether the level of DNA methylation was responsible for the increased BH4 levels in the urine, the degree of methylation of the promoter of the genes involved in BH4 biosynthesis was assessed. Figure 5 shows that the percentage of methylation of the promoters for *Dhfr* (Figure 5a), *Spr* (Figure 5b), and *Ptps* (Figure 5c) was not different between the groups.

**Figure 6:**
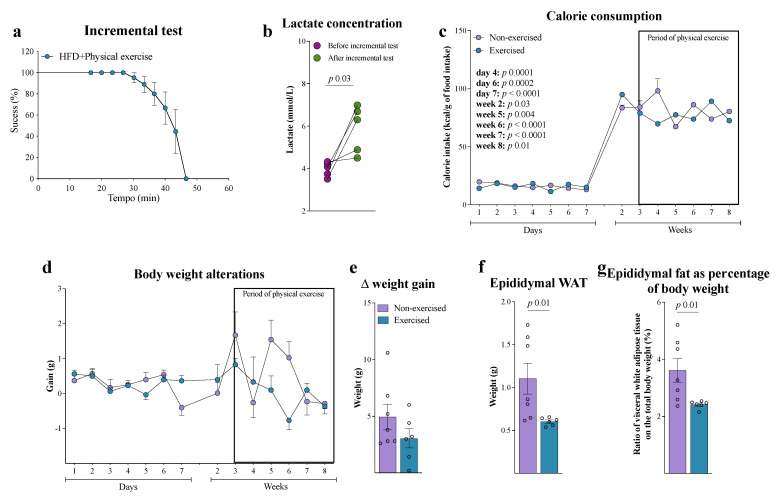
**Physical exercise reduced epididymal white adipose tissue (WAT) and body fat in C57BL/J6 mice submitted to the high-fat diet (HFD).** Adult male C57BL/J6 mice received the HFD ad libitum for 8 consecutive weeks (proteins: 12 kJ% (19 g%); carbohydrates: 27 kJ% (45 g%); lipids: 61 kJ% (36 g%)). After 2 weeks of the dietary intervention, a group of mice was also submitted to physical exercise 5 times per week for 6 weeks (for details see M&M). (**a**) The incremental test. (**b**) The caudal blood lactate concentration before and after the incremental test. (**c**) Calorie intake and (**d**) body weight gain were assessed daily for the first week of the treatment, and weekly for the next 7 weeks. (**e**) The delta of weight gain between the final weight and the initial weight. (**f**) Epididymal WAT in grams after 8 weeks of interventions (8 weeks of dietary intervention, plus 6 weeks of concomitant physical exercise). (**g**) The relationship between epididymal WAT and body weight. ANOVA for repeated measures followed by the Šídák post hoc test for multiple comparisons for (**a**,**b**) the one-tailed Student’s *t*-test for (**c**–**e**). (n = 6–7 animals per group).

The HFD-fed mice were also submitted to moderate-intensity physical exercise for 6 weeks, starting 2 weeks after initiating the dietary intervention. Figure 6 shows the effects of physical exercise on caloric intake, body weight, and eWAT accumulation. Figure 6a illustrates the results of the incremental test, represented by the percentage of success. Figure 6b shows increased lactate concentrations after the incremental test, demonstrating exhaustion. As shown in Figure 6c, caloric consumption was higher after week 2, 5, and 7 in the exercised group, although no differences were observed in the percentage of weight gain (Figure 6d) and in the weight gain delta (Figure 6e). Physical exercise prevented the increase in eWAT weight [*t*_(11)_ = 2.56; *p* ≤ 0.05] (Figure 6f) and body fat [*t*_(11)_ = 2.64; *p* ≤ 0.05] (Figure 6g) after 6 weeks of the physical exercise intervention. Supplementary Figure S3A,B are representative images from a mouse from each experimental group.

**Figure 7:**
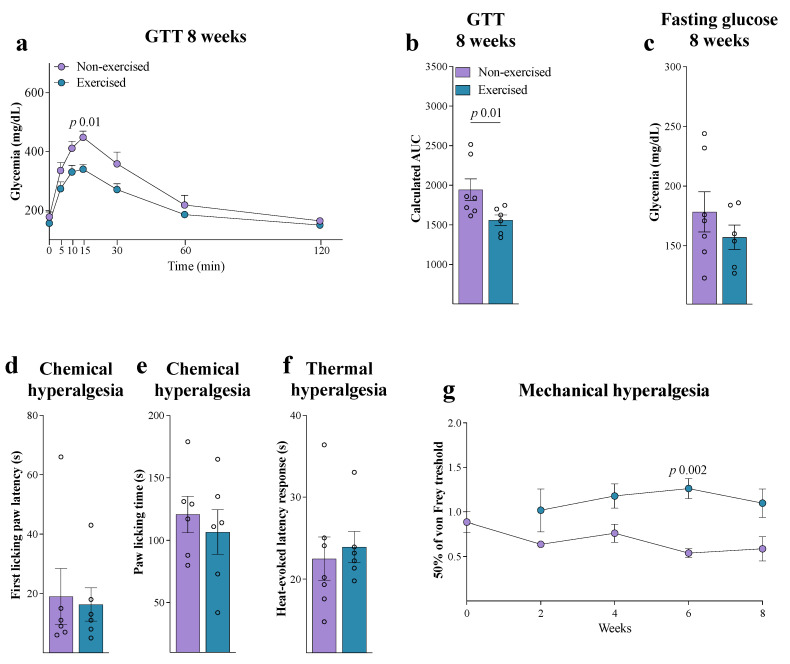
**Physical exercise normalized the glucose tolerance test and hyperalgesia evoked by mechanical stimuli in C57BL/J6 mice submitted to the high-fat diet (HFD).** Adult male C57BL/J6 mice received the HFD ad libitum for 8 consecutive weeks (proteins: 12 kJ% (19 g%); carbohydrates: 27 kJ% (45 g%); lipids: 61 kJ% (36 g%)). After 2 weeks of the dietary intervention, the mice were also submitted to physical exercise 5 times per week for 6 weeks (for details see M&M). (**a**) The glucose tolerance test (GTT), (**b**) area under the curve of the GTT, and (**c**) fasting glucose were measured after the interventions (week 8). (**d**) The first response latency, and (**e**) total response time evoked by an injection of capsaicin in the right paw (for details see M&M). (**f**) The latency for the first response evoked by the heat of the hot plate at 50 °C. (**g**) A total of 50% of the von Frey threshold used to determine mechanical hyperalgesia evaluated before and at weeks 2, 4, 6, and 8 after the interventions. ANOVA for repeated measures followed by the Šídák post hoc test for multiple comparisons for (**a**,**g**) the one-tailed Student’s *t*-test for (**b**–**f**). (n = 6–7 animals per group).

Figure 7 shows the effects of physical exercise on the GTT and nociceptive parameters after the interventions, diet plus exercise. Figure 7a shows that the GTT was significantly decreased after 15 min of the glucose challenge in the exercised mice submitted to the HFD [*F*_(6,66)_ = 2.74; *p* ≤ 0.05]. Similarly, the area under the curve was also reduced [*t*_(11)_ = 2.41; *p* ≤ 0.05] (Figure 7b). However, fasting glucose (Figure 7c) was not different between the groups at the end of the interventions. When the nociceptive thresholds were assessed, no differences were observed in (Figure 7d) chemical or thermal hyperalgesia after the 8-week dietary intervention (Figure 7e). However, mechanical hyperalgesia was reduced at week 6 (Figure 7g) in the exercised mice submitted to the HFD [*t*_(11)_ = 18.21; *p* < 0.01].

## 4. Discussion

Obesity is a chronic disease that arises directly from the impact of excess body fat on the functioning of organs and tissues, in general associated with the increased consumption of dietary saturated fats and a lifestyle with more sedentary behavior [1]. HFD-fed mice are commonly used to study potential treatments or the physiopathology of obesity because the induced metabolic alterations are like those seen in individuals affected by the disease [39]. Here, we showed that an HFD promoted eWAT accumulation, increased the eWAT to total body weight ratio, and increased body weight in adult male C57BL/J6 mice. The energy efficiency of the diets showed higher weight gain per calorie intake in the HFD-fed mice. However, the weekly food consumption showed that, on average, 24 g of the standard diet and 13 g of the HFD were consumed, resulting in 90 kcal/g and 80 kcal/g, respectively. That resulted in a slightly lower caloric intake in the HFD group.

Obesity is evidenced by adiposity in visceral and subcutaneous WATs [39]. Considering that lipids are significant sources of energy and that adipocytes have great potential for hypertrophy and hyperplasia in response to fat ingestion, the saturated fat in an HFD plays a fundamental role in the formation of large deposits of body fat [40]. Our results agree with other studies that fed mice with an HFD and observed increased body weight gain, epididymal fat accumulation, and changes in the lipid profile [41].

The adipose tissue is a complex, essential, and highly active metabolic and endocrine organ that constitutes a source of hormones, peptides, cytokines, and adipokines [7]. Adipocytes in WAT release MCP-1 (monocyte chemoattractant protein-1), which attracts monocytes and favors their differentiation into pro-inflammatory polarized M1 macrophages, forming “crown-like structures” around the WAT. This mechanism is known to provoke the death of adipocytes and increased local levels of TNF-α, IL-1β, and IL-6 [42,43]. Furthermore, adipocytes tend to rupture during obesity due to the limited capacity for expansion, leading to apoptosis and, consequently, to sustained inflammation. In this scenario, it is known that the pro-inflammatory IL-1β released by adipocytes can rapidly and directly activate nociceptors and induce hypersensitivity to pain [44]. WAT is innervated by sensory neurons and presents bidirectional communication with the brain through afferent and efferent sensory fibers [45]. Adipocyte size, lipid mobilization, and paracrine secretion are controlled by WAT nerve endings [46]. Thus, sensory neurons that innervate the WAT are involved in the production of cytokines and the influx of immune cells, playing a central role in the low-grade inflammation observed in obesity [46]. The increased eWAT in mice fed with the HFD that we observed plays an important role in the development of mechanical hypersensitivity. Mechanical hyperalgesia can be triggered by the process of hypertrophy and hyperplasia of adipocytes in an inflammatory state. IL-1β is also a stimulus that massively activates BH4 synthesis via the de novo pathway in immune cells, sensitizing nociceptive fibers and contributing to pain hypersensitivity [12,14,16,18,37,47].

The relationship between BH4 and pain was first discovered through the identification of an allele of the *GCH1* gene haplotype that encodes for the GTP cyclohydrolase, the rate-limiting enzyme for BH4 biosynthesis, associated with reduced pain scores in multiple independent neuropathic pain cohorts (for review see [17]). Based on this human validation of the biological role of BH4 in neuropathic and inflammatory pain, we demonstrated that excessive BH4 levels were produced by neurons in active pain, and by immune cells infiltrating damaged nerves and inflamed tissues [16,18,37]. Based on this information, two inhibitors for BH4 production were developed (SPRi3 and QM385) and have shown that inflammation and pain hypersensitivity were reduced, along with decreased BH4 levels in targeted tissues [16,18,37]. Furthermore, we discovered that the BH4-related metabolite, sepiapterin, accumulates in tissues and fluids exposed to the inhibitors [37]. Urinary sepiapterin was also validated as a sensitive, specific, and non-invasive biomarker in a cohort of healthy humans receiving sulfasalazine, a pharmacological treatment approved by the FDA for treating inflammatory bowel diseases. Indeed, sulfasalazine was recently described to be an inhibitor of BH4 synthesis [48]. These ground-breaking findings allowed us to hypothesize that excessive BH4 levels may play a significant role in pain development.

The involvement of BH4 metabolism in animal models for inflammatory and neuropathic nociception showed a marked increase in BH4 synthesis in sensory neurons and nervous tissues, inducing hyperalgesia [18]. The data presented here show that when WAT was accumulated due to diet, the levels of BH4 increased and the threshold decreased, as evidenced by the development of mechanical and chemical hyperalgesia. Increased transcription of *GCH1* and higher levels of BH4 have previously been reported in leukocytes infiltrating injured sciatic nerves, reinforcing the contribution of the immune system in the induction and maintenance of pain induced by the pathological production of BH4 [18]. Furthermore, the present data also agree with our previous report showing increased levels of plasma neopterin, the by-product of BH4 metabolism that normally increases proportionally to BH4, in individuals affected by type III obesity [49].

BH4 is produced by macrophages that infiltrate tissues as a response to inflammation [50], and its overproduction correlates with hyperalgesia [16,18,37]. Thus, increased eWAT appears to be a favorable environment for the development of pain hypersensitivity. Indeed, the results demonstrated here showed a concomitant increase in WAT, mechanical hyperalgesia, and BH4 levels. Furthermore, urinary BH4 can be proposed as a potential easy-to-access, sensitive, and reliable biomarker of pain development, and a promising target for the control of pain hypersensitivity in obesity. The use of pharmacological inhibitors aimed at reducing high levels of BH4 provides a potential therapeutic tool in the treatment of chronic pain.

The literature has extensively demonstrated that the regular practice of moderate-intensity physical exercise is beneficial for health promotion and for decreasing the risk of death from all causes (for a review, see [46]). The Physical Activity Guidelines for Americans points out that in individuals affected by chronic diseases, the practice of moderate-intensity physical exercise for 150 min a week, or the practice of high-intensity aerobic physical activity for 75 min a week, is beneficial in improving the health of chronic illnesses affected individuals. Also, muscle-strengthening activities involving all major muscle groups, if practiced at least twice a week, provide additional health benefits [51]. The effect of this intervention is more pronounced in conditions in which the physiopathology is associated with persistent activation of the immune system [52].

It is known that the regular practice of moderate-intensity physical exercise reduces systemic inflammation. Our group demonstrated that lipopolysaccharide-induced inflammation in mice provoked increased glycemia levels in the GTT and led to an increase in the concentrations of urinary neopterin [29], a sensitive marker of immune system activation [53]. We also showed that the GTT and neopterin levels were normalized by physical exercise [29]. Additionally, we have also shown that neopterin is increased in the plasma of individuals affected by obesity type III [49], and insulin resistance-associated obesity, who also showed increased markers of inflammation [54]. Additionally, we have also shown that exercise modulated the inflammatory response induced by lipopolysaccharide. This anti-inflammatory status was characterized by reduced levels of neopterin in the urine [29].

Physical exercise has also been shown to modulate BH4 metabolism in a way that contributes to its antinociceptive effects. BH4 is a critical cofactor involved in neurotransmitter synthesis and nitric oxide production, and its overproduction has been associated with chronic pain conditions [37,47]. Moderate-intensity exercise promotes an anti-inflammatory environment that can normalize exaggerated neuroimmune signaling and reduce pathological BH4 levels [47]. This regulation of BH4 may underlie the analgesic effects of exercise, as exercise-induced reduction in inflammation limits the pathological overproduction of BH4 that sensitizes nociceptive pathways [18]. Thus, the regular practice of physical exercise positively modulates the anti-inflammatory response of the immune system, normalizing the hypersensitivity scores seen, and possibly, the pathological production of BH4 in mice with increased eWAT.

## 5. Conclusions

This study showed that increased eWAT elicited the overproduction and secretion of BH4, with consequent increased scores of chemical and mechanical hyperalgesias. Thus, urinary BH4 can be proposed as a biomarker of pain in obesity that is easy-to-access and non-invasive. Finally, physical exercise can prevent the development of metabolic diseases and chronic pain, possibly by reducing WAT accumulation, BH4 overproduction, and therefore, the associated inflammatory state.

## 6. Limitations of the Study

The exclusive use of male mice in this study may have restricted the generalizability of our findings, as males and females often exhibit distinct metabolic profiles influenced by sex hormones, genetic differences, and physiological factors. For instance, male mice typically possess a higher lean body mass, which can impact metabolic rate, energy expenditure, nutrient utilization, and fat distribution. Additionally, spontaneous locomotor activity was measured only during the initial 5 min of the animal’s placement in the open field arena. This limited observation period may not capture potential behavioral effects that could emerge over extended time frames. Furthermore, the relatively short duration of the experimental dietary intervention (8 weeks) may also represent a limitation. Although sufficient to induce adiposity and metabolic alterations, some of the non-significant outcomes observed could be influenced by the absence of longer-term metabolic and physiological adaptations that may manifest over a more prolonged dietary period.

## Figures and Tables

**Table 1:** Physical exercise protocol.

Training Weeks	Session Duration (min)	Treadmill Inclination (%)	Activity Intensity (%)
1	35	2	60
2	40	2	60
3	45	2	60
Effort test to equalize intensities			
5	35	2	60
6	40	2	60

## Data Availability

The original contributions presented in this study are included in the article/Supplementary Material. Further inquiries can be directed to the corresponding author.

## Supplementary Materials

The following supporting information can be downloaded at: https://www.mdpi.com/article/10.3390/brainsci15060646/s1, Figure S1. Total body weight alterations in C57BL/J6 mice submitted to high fat diet (HFD). Adult male C57BL/J6 mice received HFD ad libitum for 8 consecutive weeks (proteins: 12 kJ% (19g%); carbohydrates: 27 kJ% (45 g%); lipids: 61 kJ% (36 g%)). Animals in the control group received a standard rodent diet for the same period (20 kJ% (24 g%); carbohydrates: 65 kJ% (63 g%); lipids: 15 kJ% (13 g%)). Body weight gain in grams were assessed daily for the first week of the treatment, and weekly for the next 7 weeks. Two-way ANOVA for repeated measures followed by Šídák post hoc test for multiple comparisons (n= 6–7 animals per group). Figure S2. Epididymal white adipose tissue (WAT) accumulation in C57BL/J6 mice submitted to high fat diet (HFD). Adult male C57BL/J6 mice received HFD ad libitum for 8 consecutive weeks (proteins: 12 kJ% (19 g%); carbohydrates: 27 kJ% (45 g%); lipids: 61 kJ% (36 g%)). Animals in the control group received a standard rodent diet for the same period (20 kJ% (24 g%); carbohydrates: 65 kJ% (63 g%); lipids: 15 kJ% (13 g%)). (A) Representative image of a mouse from the control group submitted to regular rodent diet that shows no WAT accumulation. (B) Representative image of a mouse from the group of animals submitted to HFD that shows increased WAT accumulation. Figure S3. Epididymal white adipose tissue (WAT) accumulation in C57BL/J6 mice submitted to high fat diet (HFD) and physical exercise. Adult male C57BL/J6 mice received HFD ad libitum for 8 consecutive weeks (proteins: 12 kJ% (19 g%); carbohydrates: 27 kJ% (45 g%); lipids: 61 kJ% (36 g%)). After 2 weeks of the dietary intervention, mice were also submitted to physical exercise 5 times per week for 6 weeks (for details see M&M). (A) Representative image of a mouse from the non-exercised group submitted to HFD that shows increased WAT accumulation. (B) Representative image of a mouse from the exercised group submitted to HFD that shows no WAT accumulation.
