# Supplementary material for: Pain Hypersensitivity and Increased Urinary Tetrahydrobiopterin Levels in Mice Submitted to High-Fat Diet
Source: Brain Sci. 2025 Jun 16;15(6):646. doi: 10.3390/brainsci15060646 (PMC12190635; doi:10.3390/brainsci15060646)
Supplement: Supplementary file 1 [file brainsci-15-00646-s001.zip › brainsci-3608182-supplementary.pdf]

Supplementary Material

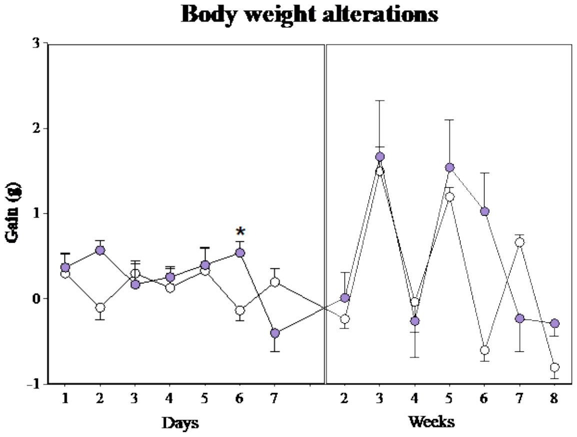

Supplementary Figure S1. Total body weight alterations in C57BL/J6 mice submitted to high fat diet (HFD). Adult male C57BL/J6 mice received HFD ad libitum for 8 consecutive weeks (proteins: 12 kJ% (19g%); carbohydrates: 27 kJ% (45g%); lipids: 61 kJ% (36g%)). Animals in the control group received a standard rodent diet for the same period (20 kJ% (24g%); carbohydrates: 65 kJ% (63g%); lipids: 15 kJ% (13g%)). Body weight gain in grams were assessed daily for the first week of the treatment, and weekly for the next 7 weeks. Two-way ANOVA for repeated measures followed by Šídák post hoc test for multiple comparisons (n= 6-7 animals per group).

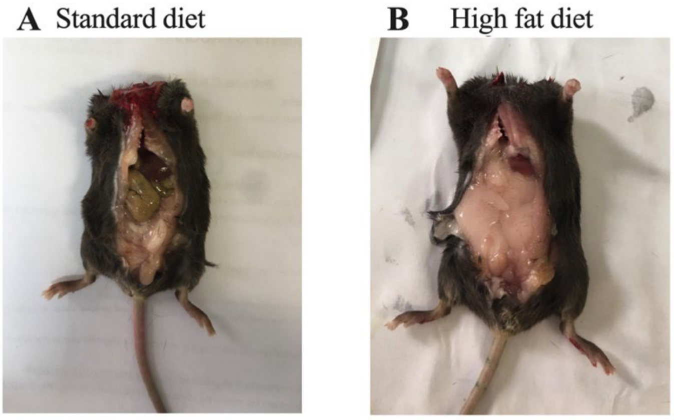

Supplementary Figure S2. Epididymal white adipose tissue (WAT) accumulation in C57BL/J6 mice submitted to high fat diet (HFD). Adult male C57BL/J6 mice received HFD ad libitum for 8 consecutive weeks (proteins: 12 kJ% (19g%); carbohydrates: 27 kJ% (45g%); lipids: 61 kJ% (36g%)). Animals in the control group received a standard rodent diet for the same period (20 kJ% (24g%); carbohydrates: 65 kJ% (63g%); lipids: 15 kJ% (13g%)). (**A**) Representative image of a mouse from the control group submitted to regular rodent diet that shows no WAT accumulation. (**B**) Representative image of a mouse from the group of animals submitted to HFD that shows increased WAT accumulation.

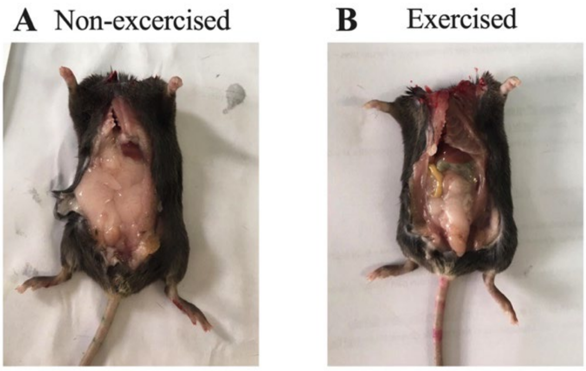

Supplementary Figure S3. Epididymal white adipose tissue (WAT) accumulation in C57BL/J6 mice submitted to high fat diet (HFD) and physical exercise. Adult male C57BL/J6 mice received HFD ad libitum for 8 consecutive weeks (proteins: 12 kJ% (19g%); carbohydrates: 27 kJ% (45g%); lipids: 61 kJ% (36g%)). After 2 weeks of the dietary intervention, mice were also submitted to physical exercise 5 times per week for 6 weeks (for details see M&M). (**A**) Representative image of a mouse from the non-exercised group submitted to HFD that shows increased WAT accumulation. (**B**) Representative image of a mouse from the exercised group submitted to HFD that shows no WAT accumulation.
